# Ribosome Protein Composition Mediates Translation during the *Escherichia coli* Stationary Phase

**DOI:** 10.3390/ijms24043128

**Published:** 2023-02-04

**Authors:** Kaspar Reier, Aivar Liiv, Jaanus Remme

**Affiliations:** Institute of Molecular and Cell Biology, University of Tartu, Riia 23b, 51010 Tartu, Estonia

**Keywords:** translation, ribosome, hibernation factors, stationary phase, ribosomal proteins

## Abstract

Bacterial ribosomes contain over 50 ribosome core proteins (r-proteins). Tens of non-ribosomal proteins bind to ribosomes to promote various steps of translation or suppress protein synthesis during ribosome hibernation. This study sets out to determine how translation activity is regulated during the prolonged stationary phase. Here, we report the protein composition of ribosomes during the stationary phase. According to quantitative mass-spectrometry analysis, ribosome core proteins bL31B and bL36B are present during the late log and first days of the stationary phase and are replaced by corresponding A paralogs later in the prolonged stationary phase. Ribosome hibernation factors Rmf, Hpf, RaiA, and Sra are bound to the ribosomes during the onset and a few first days of the stationary phase when translation is strongly suppressed. In the prolonged stationary phase, a decrease in ribosome concentration is accompanied by an increase in translation and association of translation factors with simultaneous dissociation of ribosome hibernating factors. The dynamics of ribosome-associated proteins partially explain the changes in translation activity during the stationary phase.

## 1. Introduction

Protein synthesis is crucial in all growth conditions. In a laboratory batch culture, most proteins are produced during the log phase. Later, during the late log and early stationary phase protein synthesis is downregulated. The ribosome concentration starts to decrease in the late log phase [1,2]. However, in the late log and early stationary phase translation machinery content in cells is still high, but protein production is already more than an order of magnitude lower [3]. Thus, during the early stationary phase, a ribosome subpopulation is idling.

Ribosome functioning is regulated by several ribosome-associated proteins that bind to ribosomes. In *E. coli* stationary phase, ribosome-associated proteins involve well-known ribosome modulation factor (Rmf), hibernation promoting factor (Hpf), ribosome-associated inhibitor A (RaiA), ribosome silencing factor (RsfA), energy-dependent translational throttle A (EttA), and not so well understood stationary-phase-induced ribosome-associated protein (Sra), YqjD, and YggL [4,5,6,7].

Rmf and Hpf promote conformational changes in the 70S ribosome leading to the 100S ribosome dimer formation [8]. The 100S ribosome dimer is translationally inactive but can be dissociated into functional 70S ribosomes [9,10]. RaiA and Hpf share a homologous N-terminal domain and have an overlapping binding site around the A-site of the ribosome [11,12]. The binding of RaiA to the ribosome prevents 100S formation; however, it inhibits translation by blocking the A site [12]. Two distinct roles have been proposed for Rmf, Hpf, and RaiA. First, the binding of these proteins to ribosomes causes ribosome inactivation [12]. Therefore, fewer ribosomes remain available for translation leading to its inhibition. Second, the 100S particles can stabilize ribosomes by preventing rRNA degradation by RNases [9,13,14].

During the late log phase ribosome core protein composition changes as zinc-binding r-proteins bL31A and bL36A are replaced by their corresponding paralogs bL31B (also known as YkgM, L31p) and bL36B (YkgO, L36p) lacking the Zn-binding motif via r-protein exchange [5]. Both paralogs of bL31 and bL36 bind to the same binding sites in the ribosome and can functionally replace each other [5,15]. bL31A confers higher fitness to *E. coli* than bL31B by supporting higher processivity and lower frameshift frequency of protein synthesis [5]. Both bL31 and bL36 are non-essential r-proteins as their paralogous genes (*rpmE* for bL31A, *ykgM* for bL31B, *rpmJ* for bL36A, and *ykgO* for bL31B) can be deleted either one at a time or in various combinations without the loss of viability [5,16,17,18].

During the prolonged stationary phase as defined [19], the ribosome concentration decreases gradually [2]. Ribosome small subunits are turned over faster as compared to the large subunits [2]. In contrast, only a specific set of 20 r-proteins (short-lived r-proteins) are degraded concomitantly with rRNA. More than half of r-proteins remain in the proteome for at least 14 days as free proteins (stable r-proteins) [2]. 70S ribosomes contain equimolar amounts of ribosomal core proteins during the prolonged stationary phase [2]; however, proteins bL31 and bL36 were not studied under those conditions because underlying heterogeneity of reference ribosomes. Additionally, ribosome-associated proteins dynamics have not been studied during the prolonged stationary phase. These two aspects are important for a detailed understanding of translation and its control during the stationary phase, which reflects bacterial life under natural conditions.

Here, we report the presence of the A and B paralogs of ribosomal core proteins bL31 and bL36 in the ribosomes during the stationary phase and prolonged stationary phase. Ribosome hibernation factors Rmf, Hpf, and RaiA are found in the ribosome fraction during the first days of the stationary phase. Rmf is degraded after a few days in the stationary phase when 100S ribosome dimers disappear. Hpf and RaiA are stable in the proteome, but their level in the ribosomes decreases along the progression of the stationary phase. The results are compatible with the previously proposed model [3], where depositing ribosomes in the form of hibernating particles, is important for maintaining the translation elongation rate.

## 2. Results

### 2.1. Translation Activity during the Stationary Phase

The decrease in translational activity during the onset of the stationary phase is a known phenomenon. To elucidate how and ultimately why the translation activity changes during the stationary phase, the translation activity in the stationary phase was determined. The translation activity of *E. coli* was robustly estimated by analysis of the incorporation of radioactively labeled amino acids into peptides. *E. coli MG1655-SILAC* strain was grown in MOPS minimal medium. At the mid-log phase, days 1, 2, 4, 6, 8, 10, 12, and 14, a sample of cell culture was pulsed with [^14^C] labeled amino acids. Aliquots were taken every 30 min and [^14^C] label amount in the proteins was determined in the TCA insoluble material in a liquid scintillation counter. The radioactivity was normalized against the A_600_ of the cell culture at a given time point. The incorporation of labeled amino acids into proteins over 3 h of incubation was determined (Figure S1).

**Figure 1:**
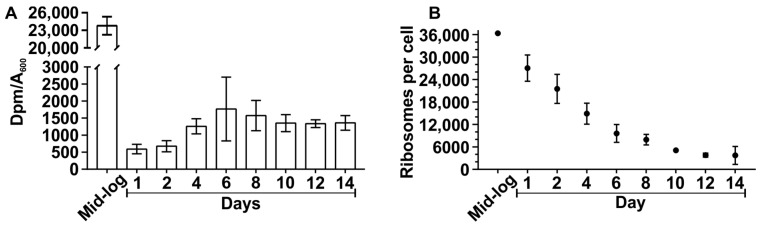
Translation activity during the stationary phase. *E. coli* culture was grown for 14 days. A radioactive isotope-labeled amino acid mix was added to a portion of the culture at designated time points. Samples were taken every 30 min over 3 h of incubation. Samples were TCA precipitated and the incorporation of the radioactive label over time was measured. Obtained values of disintegrations per minute (DPM) were normalized against A_600_ and plotted (see Supplementary Figure S1). (**A**) To evaluate changes in translation activity for 14 days, dpm/A_600_ after 60 min of incubation from each time point is shown in the figure. (**B**) The NRC during the stationary phase. Total RNA concentration was determined in the stationary phase and normalized to the mid-log timepoint. The NRC value was calculated, as described in the methods. Values shown in the figure are the mean of three independent biological experiments with standard deviation (n = 3; mean ± SD).

Translation activity was estimated according to the amount of [^14^C] label per A_600_ unit after 60 min of incubation. It comes as no surprise that translation activity in the cell culture decreases 40 times between mid-log phase and day 1 (Figure 1A). However, as cells stay under stationary phase conditions, the translation activity increases by two times between day two and four (Figure 1A). Translation activity was further confirmed via upshift in translation factors and RNA polymerase subunits in the ribosome fraction (Figure S3). These results show that translation activity is substantially decreased as cells enter the stationary phase but is also followed by a noticeable increase after the initial response.

The translation activity is strongly dependent on the number of active ribosomes, hence the question of how the ribosome population changes during the stationary phase in response to changes in translation activity was asked. The number of ribosomes per cell (NRC) was determined according to the total RNA extracted from a mass unit of cells assuming that 80% of total *E. coli* RNA is rRNA [2,20]. Using the connection between log phase generation time and the number of ribosomes per cell from [21], the NRC value was calculated, as described in the methods. It is noteworthy that the NRC decreases gradually while the translation level drops stepwise 40 times upon entry into the stationary phase (Figure 1B) resulting in a large fraction of non-translating ribosomes.

### 2.2. Translation Hibernation in Stationary Phase by Hibernation Factors

Previous studies have reported the formation of translationally inactive 100S ribosome dimers during the early stationary phase [4,22]. Indeed, we observed an accumulation of 100S ribosome dimers according to sucrose gradient centrifugation analysis during the early stationary phase (Figure S2). A decrease of at least 7 times in 100S level was observed on day four when translation activity had increased compared to day one (Figure 1A) and NRC decreased by 50% (Figure 1B and Figure S2). Thus, a large number of idling ribosomes correlate with the 100S particle accumulation.

To understand the dynamics of ribosome hibernation, ribosome-associated proteins were quantified in the stationary phase. Proteins associated with ribosomes and in total proteome were analyzed by quantitative mass spectrometry (qMS), as described in [2]. Briefly, *E. coli MG1655-SILAC* cells were first grown in “heavy” Arg and Lys containing MOPS medium until the mid-log phase followed by the addition of unlabeled (“light”) Arg and Lys in 20-fold molar excess. Samples were collected over the course of 14 days. Growth parameters (A_600_, number of viable cells) are described in [2]. Ribosomes were isolated by sucrose gradient centrifugation, as specified in the Materials and Methods. Proteins were isolated from ribosome fraction and total cell extract, fragmented, and quantified by tandem mass spectrometry (LC-MS/MS). The relative quantities of proteins were ascertained and normalized to the corresponding values of day one.

**Figure 2:**
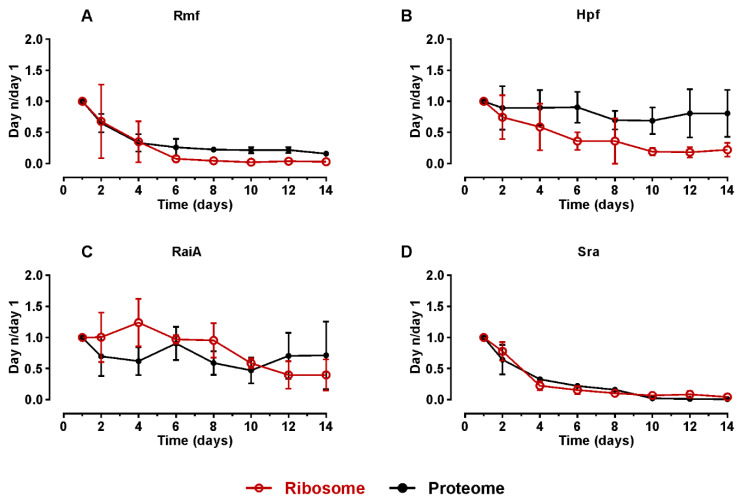
Translation hibernation factors in *E. coli* stationary phase ribosomes and proteome. *E. coli* batch culture was grown and samples were collected over the course of 14 days. Total protein and isolated ribosomes from the cell culture were analyzed using qMS. Quantity of Rmf (**A**), Hpf (**B**), RaiA (**C**), and Sra (**D**) in isolated ribosomes (red, empty) and proteome (black, filled) during stationary phase. Relative protein abundance compared to respective values on day 1 is shown (Y-axis). Values shown are the mean of three independent biological experiments with standard deviation (n = 3; mean ± SD).

Known ribosome hibernation factors (Rmf, Hpf, and RaiA) were quantified in the ribosome fraction (the 70S and 100S combined) and in the total proteome using the qMS approach. At the beginning of the stationary phase (day 1) factors Rmf, Hpf, RaiA, and Sra are bound to the ribosomes. Compared to day 1, Rmf quantity decreases gradually in ribosomes until day 6. After 6 days, the level of Rmf was below the detection limit in the ribosomes (Figure 2A), which was the same in the proteome—decreases until day 6 and after that not found in detectable amounts (Figure 2A). The degradation of Rmf explains the reduction in 100S particle levels as this factor is essential for 100S formation in *E. coli* [8,12]. Protein Hpf levels also drop at least 50% by day 6 in ribosome fraction (Figure 2B). Hpf level in the proteome remains unchanged for 14 days indicating that Hpf is a stable protein. The quantity of RaiA bound to the ribosomes gradually decreases to 40% on day 12. By day 14, about 40% of RaiA is still present in ribosomes, as compared to its content on day one (Figure 2C). In the proteome, RaiA quantity shows no change in 14 days; hence, it is a stable protein such as Hpf. Protein Sra is also present in the ribosomes at the early stationary phase but disappears from the ribosome and proteome fractions such as Rmf (Figure 2D). This raises the possibility that Rmf and Sra are functionally connected. RaiA and Hpf, the proteins sharing a homologous domain, are stable and associate with the ribosome at a significant level for 14 days. In conclusion, ribosome hibernation is a phenomenon present during the onset and early stages of the stationary phase but is gradually lost as translation activity increases.

### 2.3. bL31 and bL36 Paralogs in Stationary Phase Ribosomes

**Figure 3:**
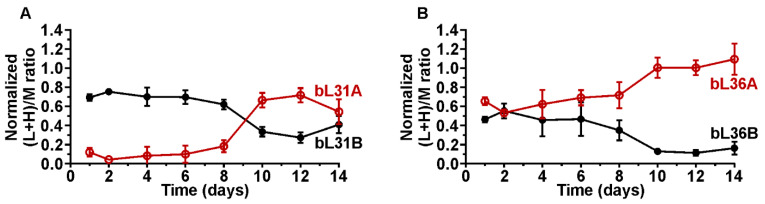
bL31 and bL36 paralogs in *E. coli* stationary phase ribosomes. *E. coli* batch culture was grown and samples were collected over the course of 14 days. 70S ribosomes were isolated and mixed in a 1:1 ratio with medium-heavy labeled reference 70S ribosomes for r-protein quantification using LC-MS/MS. The relative quantities of bL31A and bL36A (red, empty), and bL31B and bL36B (black, filled) are presented as the (L + H)/M ratio (L + H = sample; M = reference), and normalized against the average of (L + H)/M ratio of all 50S r-proteins and respective r-protein fraction in the reference ribosomes (see materials and methods). (**A**) Quantity of bL31A and bL31B in 70S ribosomes during 14 days of stationary phase. (**B**) Quantity of bL36A and bL36B in 70S ribosomes during 14 days of stationary phase. Values shown are the mean of three independent biological experiments with standard deviation (n = 3; mean ± SD).

Previous studies have shown that during the early log phase ribosomes contain bL31A and bL36A that are exchanged against bL31B and bL36B, respectively, when *E. coli* cell culture transitions into the stationary phase [5]. To understand the importance of bL31 and bL36 paralogs in ribosomes during the stationary phase, the quantities of bL31 and bL36 paralogs were determined in ribosomes isolated from stationary phase culture. The bL31A and B alongside bL36A and B were quantified in stationary phase ribosomes via qMS, as specified in the methods section. The normalized (L + H)/M ratio was calculated for each bL31 and bL36 paralogue in stationary phase ribosomes (Figure 3). (L + H)/M ratio compares relative quantities of r-proteins in the stationary phase samples (L + H) to the reference ribosomes (M).

During the first 8 days, bL31B is more abundant in the ribosome than bL31A (Figure 3A). However, after 8 days the quantity of bL31A increases and bL31B decreases in the ribosomes. At the end of 14 days, bL31A is slightly more abundant in the ribosomes than bL31B. In contrast, the quantity of bL36A and bL36B is similar in the ribosomes during the first 2 days of the stationary phase. Starting from day 2, bL36A starts to gradually increase in the ribosomes, while bL36B decreases. On day 14, the majority of ribosomes contain bL36A and a small fraction contains bL36B. Based on these results, bL31B and bL36B are exchanged against bL31A and bL36A in the ribosomes during the prolonged stationary phase.

To find out whether the bL31A and bL36A are stable or short-lived r-proteins, their quantities were determined in the early stationary phase proteome (Figure S4). The quantity of both bL31A and bL36A decreases in the early stationary phase, indicating that they are degraded (short-lived r-proteins) if not bound to the ribosomes (Figure S4). In conclusion, the newly synthesized bL31A and bL36A are incorporated into fully assembled stationary phase ribosomes in the later stages of the stationary phase as translation activity increases.

## 3. Discussion

*E. coli* ribosomes were shown to be heterogenous during the log phase and early stationary phase in respect of core ribosome proteins bL31 and bL36 [5]. Both proteins have two paralogs A and B. bL36B was shown only recently to be a true ribosome core protein that binds to the same site as bL36A [5]. Here, we demonstrate that bL31B and bL36B are replaced in the ribosome during the stationary phase increasing the level of heterogeneity of the ribosome population. In addition, ribosome-associated protein pattern changes during the progression of the stationary phase.

**Figure 4:**
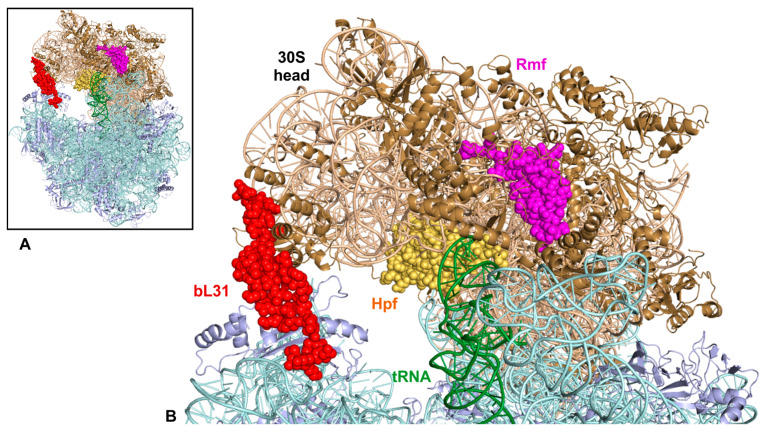
Structure of Rmf and Hpf bound to 70S ribosome. (**A**) Overall view of the complex. Rmf (magenta), Hpf (yellow), and bL31 (red) are shown as spheres. rRNA and proteins of the ribosome subunits are shown as cartoons in either teal and light blue (50S) or wheat and sand (30S) colors, respectively. tRNA bound to ribosome A-site is shown as cartoon and colored green. (**B**) detailed view of 50S and 30S interface between 50S central perturbance and 30S head region. The model for the Rmf–Hpf–70S was obtained from a published cryo-electron microscopy structure of the *E. coli* 70S particle in complex with Rmf, Hpf, and an A-site tRNA (PDB ID: 6H4N) [27].

Ribosomal core protein bL31 is the main component of the ribosomal intersubunit bridge B1b [23,24] (Figure 4). bL31 is important for the stable interaction of ribosome subunits, it facilitates translation initiation, and plays an important role in maintaining apparent translation processivity and reading frame maintenance during translation [15,16,17]. In *E. coli,* bL31 is encoded by two paralogous genes, *rpmE* (bL31A) and *ykgM* (bL31B) [25]. The sequence identity between the two paralogous proteins is 35% but the 3D structure is similar (5). bL31A has two cysteine-containing Zn fingers which bind one Zn^2+^ ion [26]. bL31A confers higher fitness to bacteria, higher apparent translation processivity, and lower frameshift frequency, as compared to bL31B [17]. On the other hand, bL31B has an extra loop between two domains of the protein. The extra loop can potentially mitigate the extensive 30S head rotation needed for 100S ribosome dimer formation [12], as suggested in [5]. Notably, bL31 is essential for 100S ribosome dimer formation. Both bL31A and bL31B can support ribosome dimerization [15].

In addition, bL31B is hypothesized to be more resistant to oxidation, as compared to bL31A, as the A paralog contains four cysteine residues, and the B paralog does not. Therefore, the observation that the ribosomes of the early stationary phase contain preferentially bL31B can be rationalized by tolerance to oxidative stress. bL31A is the dominant form in the log phase ribosomes [5] when protein synthesis is most active and its level increases again during the prolonged stationary phase.

Little is known about the functional role of bL36 in the ribosomes. Deletion of *rlmJ* (bL36A) leads to slow growth phenotype at 25–42 °C and alterations in the reactivity of specific 23S rRNA bases [28]. Lack of bL36 in the ribosomes was shown to reduce translation in vitro by 30% [15]. Both paralogs of bL36 share an identical location in an RNA pocket and are not exposed to the ribosome surface [5]. It is evident that the bL36 paralogs are replaced on the existing ribosomes as ribosome synthesis does not occur during the stationary phase. The exchange of bL36 paralogs in the ribosome is a steric challenge and would need a specific catalyst. Dissociation of bL36 from the 23S rRNA pocket needs extensive structural rearrangements which are likely catalyzed by an exogenous and yet unidentified factor, as suggested earlier [29,30].

bL31A and bL36A are synthesized during the stationary phase according to their isotopic content. This raises the question about the regulation of *rpmE* and *rpmJ* genes. The monocistronic operon *rpmE* is expressed from two promoters [31]. The expression of bL31A is under translational feedback control by bL31A binding to a stem of its own mRNA but not to the *YkgMO* mRNA encoding bL31B and bL36B [31]. A recent paper demonstrates that both bL31A and bL31B can repress the translation of bL31A by a similar mechanism [32]. It is possible that one of the two *rpmE* promoters is needed for the expression of bL31A during the stationary phase.

bL36A gene *rpmJ* is the last member of a large *spc* operon encoding 11 r-proteins and a membrane translocase subunit SecY as a penultimate cistron. First members of the *spc* operon are under feedback translational control by r-protein uS8, a mechanism shared by many r-proteins [33]. However, the regulation of bL36A expression has not been analyzed. Other r-proteins of the *spc* operon (uL14, uL24, uL5, uS14, uS8, uL6, uL18, uS5, uL30, and uL15) do not appear to be synthesized during stationary phase [2]. Thus, the *rpmJ* gene is expected to be expressed from a yet unidentified promoter during the stationary phase.

bL31B and bL36B are encoded by the *ykgMO* operon, which is under the control of Zur (Zn uptake regulator) repressor. Zur binds to its target sites in the presence of Zn^2+^. Expression of the *ykgMO* operon during the transition to the stationary growth phase cannot be explained by decreases in Zn^2+^ concentration in the culture medium as the bL31B and bL36B levels change during the stationary phase while Zn^2+^ remains constant. Again, an alternative regulation mechanism is expected to control the expression of bL31B and bL36B during the transition to the stationary phase.

During the transition to the stationary phase, the translation activity of the *E. coli* culture is reduced by 40 times while the NRC is still high (Figure 1). (p)ppGpp-dependent regulation is mostly responsible for the downregulation of stable RNA and housekeeping genes and the activation of stress-related genes [34]. A large fraction of idle ribosomes is in part in the form of 100S translationally inactive ribosome dimers. The formation of ribosome dimers depends on the binding of Rmf and Hpf [22]. 100S particles are known to disappear after some time in the stationary phase depending on the medium [4]. At the same time, when 100S ribosome dimers are abundant, RaiA and Sra bind to the ribosomes in addition to Rmf and Hpf (Figure 2 and Figure S2). RaiA binds to the 70S ribosomes to induce translationally inactive conformation [12]. We have found that Rmf and Sra dissociate from the ribosomes and disappear from the proteome before day 6 of the stationary phase (Figure 2A, D). It is tempting to speculate that Sra has a role in Rmf functioning as these two proteins bind to the ribosomes and are degraded in concert. Ribosome hibernation factors Hpf and RaiA are stable proteins and are found in the ribosomes during prolonged stationary phase albeit at a low level (Figure 2B,C). During prolonged stationary phase (after day 6) translation appears to be activated again as evidenced by association of translation factors EF-Tu, EF-Ts, EF-G, IF2, and RNA polymerase subunits with the ribosomes (Figure S3).

The physiological importance of ribosome hibernation is still disputable. This study and others [35] demonstrate that during the transition to the stationary phase and under other stress conditions translation is silenced by a specialized set of factors. An important aspect is the economy in cellular energy consumption as nearly 40% of energy is used by translation in the log phase. However, specific sets of proteins are synthesized under stress conditions. The presence of a large fraction of non-translating ribosomes and a low level of GTP can reduce the translation rate of individual proteins. An elegant study [3] demonstrated that reducing the number of translation-competent ribosomes maintains the translation elongation rate under energy deficiency. The translation elongation rate is important for peptidyl-tRNA-dependent regulation of translation and co-translational folding of proteins. We speculate that one function of ribosome hibernation during the transition from log to stationary phase and the early stationary phase is titrating ribosomes away from translation to maintain polypeptide elongation rate.

## 4. Materials and Methods

### 4.1. Measurement of Translation Activity during the Stationary Phase

*E. coli MG1655-SILAC (F-, λ-, rph-1, ΔlysA, ΔargA)* strain, which is incapable of de novo synthesis of arginine and lysine, was grown in MOPS medium [36]. At mid-log (A_600_ ≈ 1), 2 mg/mL of unlabeled arginine (Arg0) and lysine (Lys0) were added to the culture. Cell culture was grown for a maximum of 14 days. A portion of the cells were collected in the mid-log phase and on days 1, 2, 4, 6, 8, 10, 12, and 14 after the start of the experiment. Cells were pulse-labeled with 0.5 mCi of [^14^C]-labeled amino acids (L-Amino acid mixture; Hartmann Analytic) and incubated at 37 °C on a shaker for 3 h. Samples were collected every 30 min and precipitated with TCA (trichloroacetic acid). Radioactivity in the precipitate was determined using an Optiphase HiSafe III scintillator (PerkinElmer). Disintegrations per minute (Dpm) values were normalized to the A_600_ of the cell culture at a respective timepoint. The experiment was performed in three biological replicates.

### 4.2. Determining the Number of Ribosomes per Cell (NRC)

The *E. coli MG1655-SILAC (F-, λ-, rph-1, ΔlysA, ΔargA)* strain was grown in an MOPS medium. At mid-log (A_600_ ≈ 1), 2 mg/mL of unlabeled arginine (Arg0) and lysine (Lys0) were added to the culture. Cell culture was grown for a maximum of 14 days. Samples were collected in the mid-log phase (A_600_ ≈ 1) and on days 1, 2, 4, 6, 8, 10, 12, and 14 after the start of the experiment. Total RNA was extracted from the cells, as described in [2]. RNA concentrations (A_260_) were normalized by dividing with the mid-log phase A_260_ value (day n/mid-log). Log phase generation time and the number of ribosomes per cell from [21] were used for the creation of equation: Y = 28371x − 12289, where Y is the number of ribosomes per cell and X is the generation time (hours). Using the generation time of the *E. coli MG1655-SILAC* strain used in this work (average 35 min), the NRC in the mid-log phase was calculated. Multiplying the day n/mid-log value in the stationary phase with NRC in the mid-log phase allowed the estimation of NRC in stationary phase cells.

### 4.3. Cell Cultivation and Ribosome Isolation for Mass Spectrometry Analysis

The *E. coli MG1655-SILAC (F-, λ-, rph-1, ΔlysA, ΔargA)* strain was grown in a MOPS medium [36], supplemented with heavy labeled arginine (Arg10–[^13^C]_6_H_14_[^15^N]_4_O_2_) and lysine (Lys8–[^13^C]_6_H_14_[^15^N]_2_O_2_) (SILANTES, Germany). At mid-log (A_600_ ≈ 1), 2 mg/mL of unlabeled arginine (Arg0) and lysine (Lys0) were added to the culture. Cultures were grown for a maximum of 14 days. Samples were collected on days 1, 2, 4, 6, 8, 10, 12, and 14 by low-speed centrifugation.

As an internal reference, *E. coli MG1655-SILAC (F-, λ-, rph-1, ΔlysA, ΔargA)* strain was grown in MOPS medium supplemented with 0.1 mg/mL medium-heavy arginine (Arg6–[^13^C]_6_H_14_N_4_O_2_) and lysine (Lys4–C_6_H_10_[^2^H]_4_N_2_O_2_) (SILANTES, Germany). Cells were grown to mid-log (A_600_ ≈ 1) and harvested by low-speed centrifugation (4500 g/15 min).

Ribosomes were isolated from the samples, as described in [2]. Whole proteome and ribosome protein composition were analyzed via qMS, as described in [2].

### 4.4. Quantification of Ribosome-Associated Proteins

Data analysis was performed using Maxquant (version 2.14) with default settings [37], except that the minimal peptide length for the specific and nonspecific search was 5 amino acids. Unique peptides were used for quantification, the main search peptide tolerance was 8 ppm, and variable modification was used for the quantitation of oxidation (methionine). The peptide identification search was carried out against the *E. coli K-12 MG1655* protein sequence database from UniprotKB (as of Mar. 2021). The search results were filtered and transformed using Perseus (v1.6.14.0) [38]. Calculations with data are listed: first, r-protein medium-heavy intensities were normalized to day 1 values and an average was calculated for each sample (=R). Next, the light (unlabeled) and heavy intensities of proteins of interest in samples were added together (L + H) and also normalized to day 1 values (L + H fraction). Finally, the (L + H) fraction for proteins of interest was divided with the R to get a normalized (L + H) fraction.

### 4.5. Quantification of r-Protein bL31 and bL36 Paralogs in Ribosomes

Data analysis was performed using Maxquant (version 2.14) with default settings, except that the minimal peptide length for the specific and nonspecific search was 5 amino acids. Unique peptides were used for quantification, the main search peptide tolerance was 8 ppm, and variable modification was used for the quantitation of oxidation (methionine). The peptide identification search was carried out against the *E. coli K-12 MG1655* protein sequence database from UniprotKB (as of Mar. 2021). Next, Maxquant results were imported to Skyline (version 20.1.0.31) and peptides originating from r-proteins bL31A, bL31B, bL36A, and bL36B (Table S1) were quantified and (L + H)/M ratio (L + H = sample; M = reference) were calculated for respective proteins. The r-protein bL36A had only one peptide for quantification. The reference ribosomes were heterogeneous concerning bL31 and bL36 paralogs [5]; however, an assumption that all ribosomes in the exponential growth phase contain at least one copy of bL31A or bL31B and bL36A or bL36B, allows quantification of A and B paralogs for both bL31 and bL36 r-proteins via single reference. The (L + H)/M ratios of bL31A and bL31B alongside bL36A and bL36B were normalized against the fraction of bL31A (0,82), bL31B (0,18), bL36A (0,88), and bL36B (0,12) in the reference ribosomes [5]. Representative MS1 spectra for bL36A peptide VICSAEPK is shown on supplementary Figure S5.

### 4.6. Quantification of r-Protein bL31 and bL36 Paralogs in Proteome

The *E. coli MG1655-SILAC (F-, λ-, rph-1, ΔlysA, ΔargA)* strain was grown in an MOPS medium. Samples were collected at 3, 6, 24 (day 1), and 48 (day 2) hours after the start of the experiment. Cells were lysed, total protein from the cells was mixed in a 24:1 ratio (A_280_) with reference ribosomes and mass spectrometrical analysis was conducted as described in [2]. Two types of reference ribosomes were used: 70S ribosomes isolated from *E. coli MG1655-SILAC (F-, λ-, rph-1, ΔlysA, ΔargA)* strain with a known quantity of bL31A (82%) and bL36A (88%) (A reference); and 70S ribosomes from *MG-SILAC ΔAA + pBT-BB*, a strain containing a plasmid (*pBT-ykgM-ykgO, AmpR*, high copy expression of bL31B and bL36B) [5], with a known quantity of bL31B (100%) and bL36B (100%) (B reference).

Data analysis was performed using Maxquant (version 2.14) with default settings, except that the minimal peptide length for the specific and nonspecific search was 5 amino acids. Unique peptides were used for quantification, the main search peptide tolerance was 8 ppm, and variable modification was used for the quantitation of oxidation (methionine). The peptide identification search was carried out against the *E. coli K-12 MG1655* protein sequence database from UniprotKB (as of Mar. 2021). Next, Maxquant results were imported to Skyline (version 20.1.0.31) and peptides from r-proteins bL31A, bL31B, bL36A, and bL36B were quantified and L/M ratios were calculated for respective proteins. In the case of bL31A and bL36A, samples containing A reference were used, and for bL31B and bL36B samples, B reference were used.

## Data Availability

Proteomics data can be found in the EMBL-EBI PRoteomics IDEntification database (PRIDE).

## Supplementary Materials

The following supporting information can be downloaded at: https://www.mdpi.com/article/10.3390/ijms24043128/s1.
