# Supplementary material for: Ribosome Protein Composition Mediates Translation during the Escherichia coli Stationary Phase
Source: Int J Mol Sci. 2023 Feb 4;24(4):3128. doi: 10.3390/ijms24043128 (PMC9959377; doi:10.3390/ijms24043128)
Supplement: Supplementary file 1 [file ijms-24-03128-s001.zip › ijms-2164712-supplementary/Supplementary figure 1.pdf]

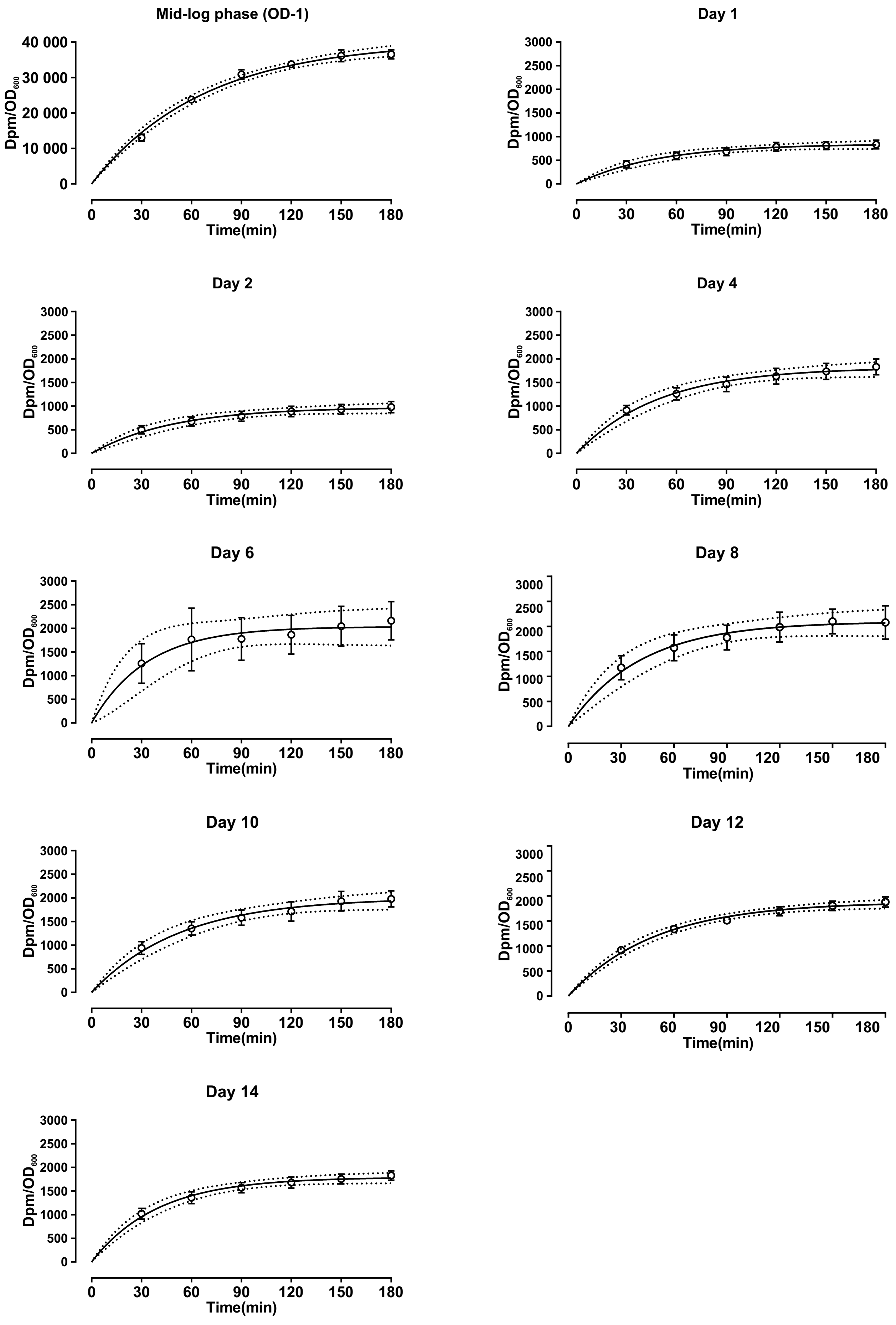
Mid-log phase (OD-1)
40 000
30 000
20 000
10 000
0
Dpm/OD600
0 30 60 90 120 150 180
Time(min)
Day 1
Day 2
Day 4
Day 6
Day 8
Day 10
Day 12
Day 14
3000
2500
2000
1500
1000
500
0
Dpm/OD600
0 30 60 90 120 150 180
Time(min)

Figure S1 – Translation activity in *E.coli* during the stationary phase. Bacterial culture was grown for 14 days. A radioactive isotope-labeled amino acids mix was added to a portion of the culture at designated time points. Samples were taken every 30 min over 3 h of incubation. Samples were TCA precipitated and the incorporation of the radioactive label over time was measured. Obtained values of disintegrations per minute (DPM) were normalized against A_{600} and plotted. Values shown in the figure are the mean of three independent biological experiments with standard deviation (n=3; mean ± SD).
