# Supplementary material for: Ribosome Protein Composition Mediates Translation during the Escherichia coli Stationary Phase
Source: Int J Mol Sci. 2023 Feb 4;24(4):3128. doi: 10.3390/ijms24043128 (PMC9959377; doi:10.3390/ijms24043128)
Supplement: Supplementary file 1 [file ijms-24-03128-s001.zip › ijms-2164712-supplementary/Supplementary figure 2.pdf]

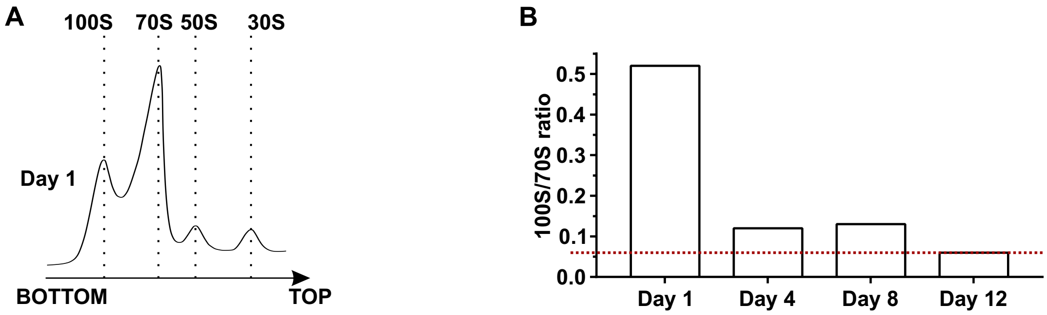

Figure S2 - 100S particles in *E.coli* during stationary phase. Culture was grown for total of 12 days. Ribosomal particles were isolated from the culture using sucrose gradient centrifugation. (A) Representative gradient profile of the day 1 culture. (B) 100S to 70S ratios calculated based on the corresponding ribosome profiles. Areas under the 100S and 70S peaks were quantified using ImageJ, and 100S/70S ratio was plotted. Baseline of minimum 100S/70S values is shown in red dotted line. Profiles used for calculation are from Reier et al. 2022 (Supplementary Figure S3).
