# Supplementary material for: Ribosome Protein Composition Mediates Translation during the Escherichia coli Stationary Phase
Source: Int J Mol Sci. 2023 Feb 4;24(4):3128. doi: 10.3390/ijms24043128 (PMC9959377; doi:10.3390/ijms24043128)
Supplement: Supplementary file 1 [file ijms-24-03128-s001.zip › ijms-2164712-supplementary/Supplementary figure 3.pdf]

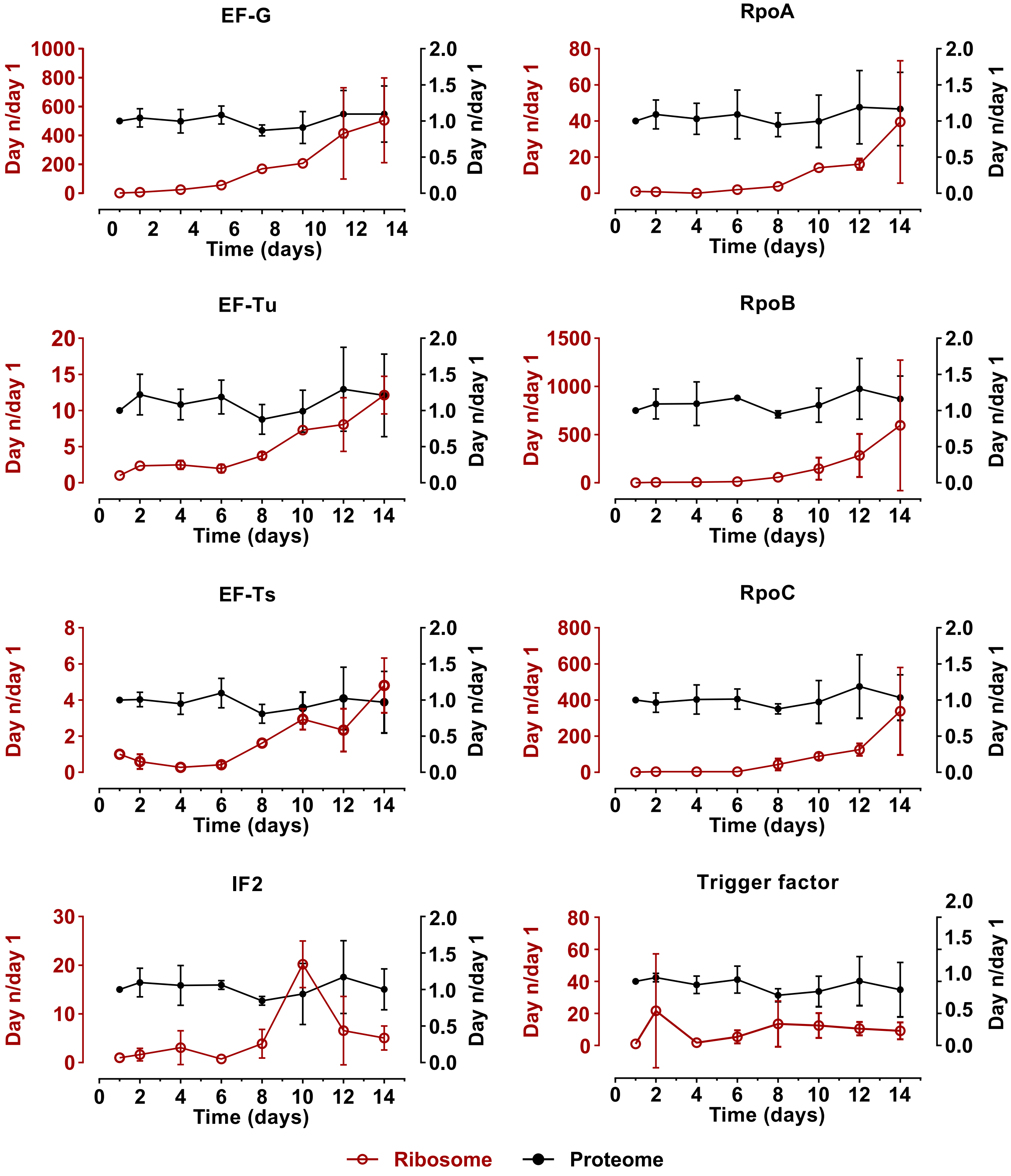

Figure S3 - Ribosome-associated proteins in *E.coli* ribosomes and proteome during the stationary phase. Samples were collected over the course of 14 days. Total protein and isolated ribosomes from the cell culture were analyzed using qMS. Relative protein abundance compared to respective values on day 1 in the proteome (black, filled) and ribosomes (red, empty) are shown (Y-axis). Values shown in the figure are the mean of three independent biological experiments with standard deviation (n=3; mean ± SD).
