# Supplementary material for: Ribosome Protein Composition Mediates Translation during the Escherichia coli Stationary Phase
Source: Int J Mol Sci. 2023 Feb 4;24(4):3128. doi: 10.3390/ijms24043128 (PMC9959377; doi:10.3390/ijms24043128)
Supplement: Supplementary file 1 [file ijms-24-03128-s001.zip › ijms-2164712-supplementary/Supplementary figure 4.pdf]

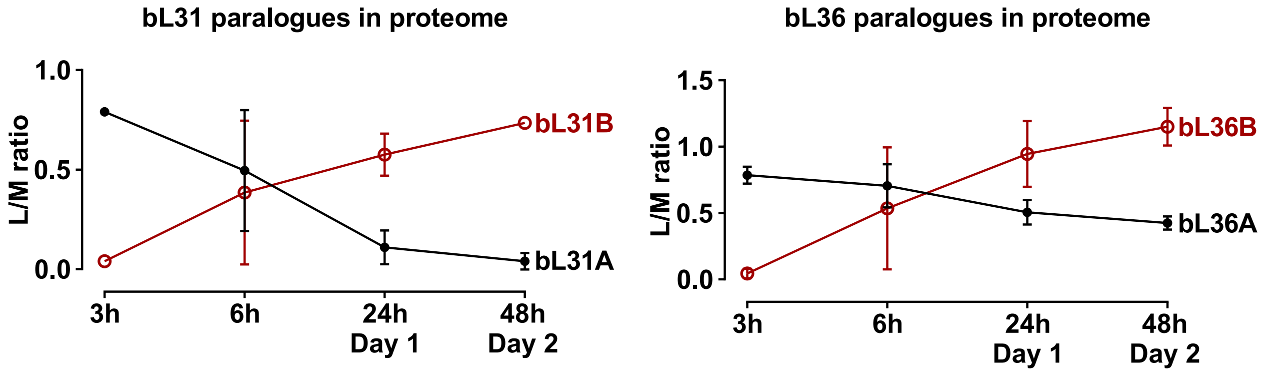

Figure S4 - bL31 and bL36 paralogues in *E.coli* early stationary phase proteome. Samples were collected over the course of two days. Total protein from the cells was mixed with reference ribosomes containing a known quantity of bL31A and bL36A (A reference) or bL31B and bL36B (B reference) and analyzed using qMS. The relative quantity of r-proteins is shown as the L/M ratio (Y-axis) and is normalized to the average L/M ratio of 50S r-proteins in the sample. Values shown in the figure are the mean of two independent biological experiments with standard deviation (n=2; mean ± SD).
