# Supplementary material for: Ribosome Protein Composition Mediates Translation during the Escherichia coli Stationary Phase
Source: Int J Mol Sci. 2023 Feb 4;24(4):3128. doi: 10.3390/ijms24043128 (PMC9959377; doi:10.3390/ijms24043128)
Supplement: Supplementary file 1 [file ijms-24-03128-s001.zip › ijms-2164712-supplementary/Supplementary figure 5.pdf]

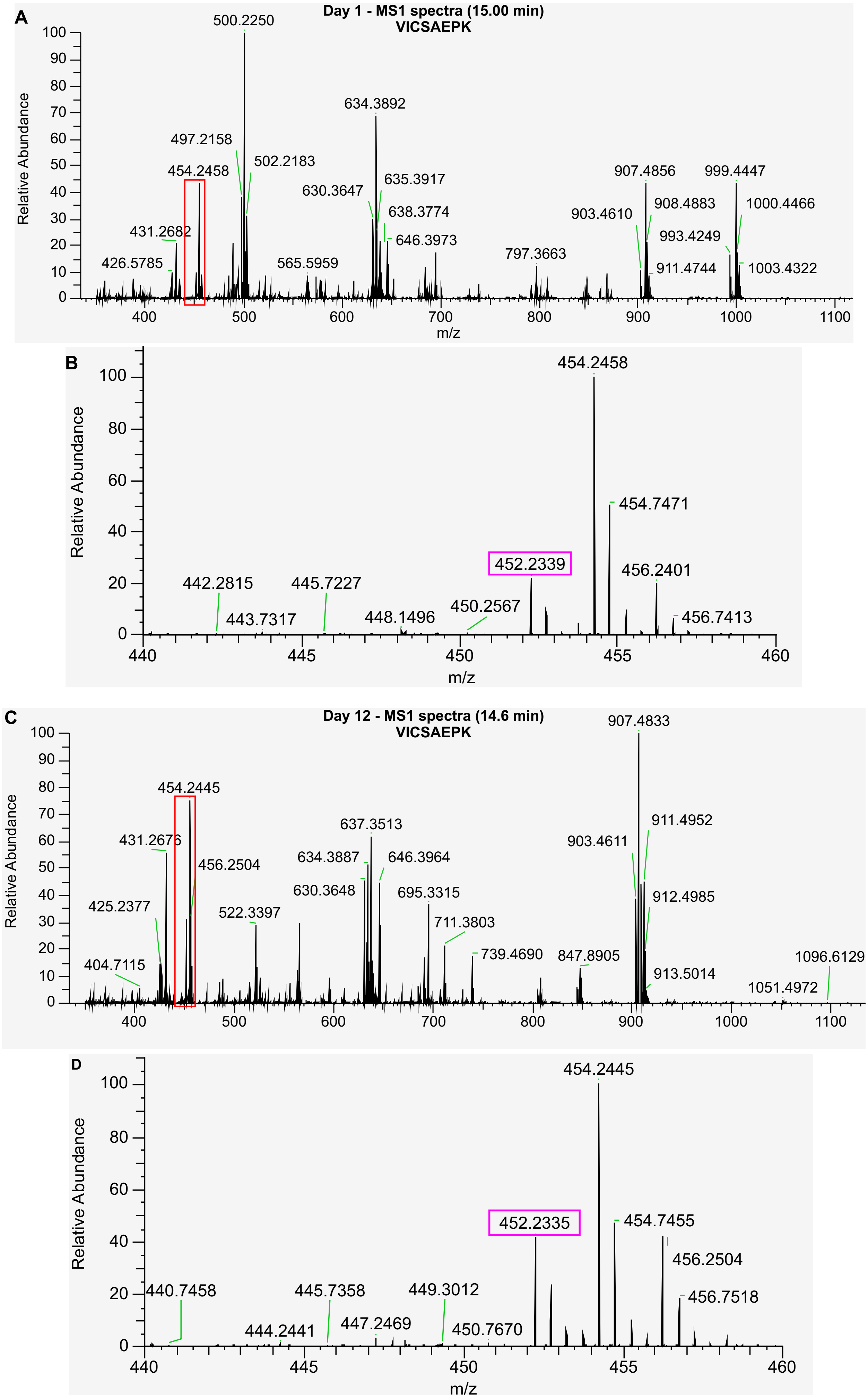
A
Day 1 - MS1 spectra (15.00 min)
VICSAEPK
Relative Abundance
m/z
500.2250
634.3892
497.2158
502.2183
454.2458
635.3917
630.3647
638.3774
431.2682
646.3973
426.5785
565.5959
797.3663
907.4856
999.4447
908.4883
1000.4466
903.4610
993.4249
911.4744
1003.4322
B
454.2458
454.7471
452.2339
456.2401
442.2815
445.7227
443.7317
448.1496
450.2567
456.7413
C
Day 12 - MS1 spectra (14.6 min)
VICSAEPK
907.4833
454.2445
911.4952
637.3513
431.2676
903.4611
634.3887
646.3964
456.2504
630.3648
695.3315
912.4985
425.2377
522.3397
711.3803
739.4690
847.8905
1096.6129
404.7115
913.5014
1051.4972
D
454.2445
454.7455
452.2335
456.2504
440.7458
445.7358
449.3012
456.7518
444.2441
447.2469
450.7670

Figure S5 - Representative MS1 spectra of bL36A peptide VICSAEPK. (A&C) Overview of MS1 spectra from day 1 and day 12 with magnified part shown in red. (B&D) Magnified view of spectra, VICSAEPK peptide shown in magenta. Spectra's shown are from LC-MS/MS with retention times 15.00 min (day 1) and 14.60 min (day 12).
