# Supplementary material for: Ribosome Protein Composition Mediates Translation during the Escherichia coli Stationary Phase
Source: Int J Mol Sci. 2023 Feb 4;24(4):3128. doi: 10.3390/ijms24043128 (PMC9959377; doi:10.3390/ijms24043128)
Supplement: Supplementary file 1 [file ijms-24-03128-s001.zip › ijms-2164712-supplementary/Supplementary Table S1.pdf]

**Table S1 - Peptide masses used for analysis from hibernation factors and bL31 & bL36 paralogs**

| Protein Name | Peptide Sequence | Ion Charge[a] | m/z (unlabeled)[a] | m/z (heavy labeled)[a] | m/z (medium-heavy labeled)[a] |
|---|---|---|---|---|---|
| bL31A | STVGHDLNLDVCSK | 2 | 772.8723 | 776.8794 | 774.8849 |
| | CHPFFTGK | 2 | 497.2342 | 501.2413 | 499.2467 |
| | VDRFNK | 2 | 389.7139 | 398.7252 | 394.7366 |
| | VDRFNKR | 2 | 467.7645 | 481.7799 | 475.7972 |
| bL31B | TVVFHDTSVDEYFK | 3 | 562.9385 | 565.6099 | 564.2802 |
| | IGSTIKTDREIELDGVTYPYVTIDVSSK | 3 | 1033.8747 | 1042.5536 | 1038.5648 |
| | TDREIELDGVTYPYVTIDVSSK | 3 | 834.0866 | 840.0941 | 837.4350 |
| | SHPFYTGK | 2 | 468.7323 | 472.7394 | 470.7449 |
| | LRTVASEGNVAR | 2 | 636.8546 | 646.8628 | 642.8747 |
| | TVASEGNVAR | 2 | 502.2620 | 507.2661 | 505.2721 |
| bL36A | VICSAEPK | 2 | 452.2339 | 456.2410 | 454.2464 |
| bL36B | VLNSLR | 2 | 351.2189 | 356.2230 | 354.2289 |
| | ERHPDCQIVKR | 4 | 360.1899 | 367.1976 | 364.2063 |
| Rmf | GYQAGIAGR | 2 | 446.7354 | * | |
| Hpf | LEQYFDR | 2 | 485.7351 | | |
| | INQVYVVLK | 2 | 538.3291 | | |
| Sra | HILGLDHK | 2 | 466.7693 | | |
| | KIVTEGDKSSVVNNPTGR | 2 | 951.0080 | | |
| | IVTEGDKSSVVNNPTGR | 2 | 886.9605 | | |
| RaiA | LAKLEK | 2 | 351.2314 | | |
| | WQTHLINPHIILSK | 2 | 850.4858 | | |
| | RAATSVKDANFVEEVEEE | 2 | 1011.9844 | | |
| | AATSVKDANFVEEVEEE | 2 | 933.9338 | | |

Footnotes:

a) Ion charge and peptide m/z values are for precursor MS1 particles

* For proteins Hpf, Rmf, Sra, RaiA heavy and medium-heavy labeled peptide were not indentified/determined
